# An Overview of Autophagy and Yeast Pseudohyphal Growth: Integration of Signaling Pathways during Nitrogen Stress

**DOI:** 10.3390/cells1030263

**Published:** 2012-07-04

**Authors:** Qingxuan Song, Anuj Kumar

**Affiliations:** Department of Molecular, Cellular, and Developmental Biology, University of Michigan, Ann Arbor, MI 48109, USA; Email: qsong@umich.edu

**Keywords:** yeast, filamentous growth, pseudohyphal growth, autophagy, PKA, Tor

## Abstract

The budding yeast *Saccharomyces cerevisiae* responds to nutritional stress through the regulated activities of signaling pathways mediating autophagy and other conserved cellular processes. Autophagy has been studied intensely in yeast, where over 30 autophagy-related genes have been identified with defined roles enabling the formation of autophagic vesicles and their subsequent trafficking to the central yeast vacuole. Much less, however, is known regarding the regulatory mechanisms through which autophagy is integrated with other yeast stress responses. Nitrogen limitation initiates autophagy and pseudohyphal growth in yeast, the latter being a fascinating stress response characterized by the formation of multicellular chains or filaments of elongated cells. An increasing body of evidence suggests an interrelationship between processes responsive to nitrogen stress with cAMP-dependent PKA and the TOR kinase complex acting as key regulators of autophagy, pseudohyphal growth, and endocytosis. In this review, we will summarize our current understanding of the regulatory events controlling these processes. In particular, we explore the interplay between autophagy, polarized pseudohyphal growth, and to a lesser extent endocytosis, and posit that the integrated response of these processes in yeast is a critical point for further laboratory experimentation as a model of cellular responses to nitrogen limitation throughout the Eukaryota.

## 1. Introduction

Organismal growth and survival is critically dependent upon the ability of cells to efficiently respond to available carbon sources, nitrogen sources, and amino acids [1,2,3]. In the budding yeast *Saccharomyces cerevisiae*, substantial cellular machinery is devoted to precisely coordinating signaling pathways that constitute the cellular response to nutrient availability. Over evolutionary time, yeast cells have developed the ability to adapt to conditions of limited nutrient availability by modulating cellular metabolism, catabolism and morphogenesis; through these mechanism, yeast cells efficiently utilize nutrients and maximize their usage. In particular, nitrogen availability and utilization is critical for the biosynthesis of genetic material, proteins, and organelles. A variety of amino acids and organic amines can be used by yeast cells as nitrogenous sources [1,4]. Under conditions of nitrogen stress or deprivation, yeast cells generate several adaptive responses, including the implementation of complex cellular programs resulting in autophagy and pseudohyphal growth. In addition, yeast cells employ endocytosis in response to cell stress to remove membrane transporters from the cell surface for trafficking to the vacuole. Collectively, these three cellular processes and their potential co-regulation in *S. cerevisiae* will serve as the focus of this review.

## 2. Autophagy and Filamentous Growth in Yeast

Autophagy is a stress-induced catabolic process by which cells recycle cytoplasm and defective organelles [5]. Multiple forms of autophagy have been identified, such as macroautophagy, microautophagy, chaperone-mediated autophagy [6], and mitochondrial autophagy [7]. Macroautophagy is a lysosome/vacuole-dependent process involving the sequestration of cytoplasmic material within specialized double-membrane vesicles for trafficking to the vacuole and subsequent degradation. By contrast, microautophagy involves the engulfment of cytosol on the surface of the vacuole by invagination of the vacuolar membrane [8]. Autophagy levels are relatively low under conditions of nutrient sufficiency but are drastically enhanced under conditions of nitrogen deprivation; nitrogen stress is a critical induction condition triggering the autophagic response. In addition, nitrogen stress can also induce morphological changes, such as filamentous growth. Filamentous growth is a morphologically distinct growth mode in many fungi wherein cells elongate and interconnect to form a multicellular “chain” [9]. In *S. cerevisiae*, the chain-like filaments are thought to allow yeast colonies to scavenge for nutrients under conditions of nutritional stress. This morphogenetic response has been observed in several fungal species, including at least two types of *S. cerevisiae* laboratory strains (e.g., Σ1278b and SK1) [10,11]; it should be noted, however, that most common laboratory strains of *S. cerevisiae* are non-filamentous (e.g., derivatives of S288c).

To date, autophagy has been extensively studied in *S. cerevisiae* and other filamentous fungi. Previous studies have indicated that yeast cells might use similar signaling pathways to regulate both autophagy and filamentous growth as adaptations to conditions of nutritional stress [12,13,14,15]. Recent research indicates that multiple autophagy-related proteins are also involved in the filamentous growth response [16,17]. Collectively, this evidence suggests a putative interrelationship between autophagy and filamentous growth in *S. cerevisiae*. Therefore in this review, we will introduce the functions of autophagy and filamentous growth in yeast, followed by descriptions of the molecular regulation of these processes and the signaling pathways that enable them. We will subsequently introduce potential sites of pathway crosstalk and interconnections, highlighting the mechanistic and regulatory commonality in both cellular responses.

### 2.1. Autophagy in Saccharomyces cerevisiae and Other Filamentous Fungi

Autophagy is known to occur in a wide range of eukaryotic organisms, from the single yeast cell to multiple differentiated cells in mammalian organisms [18]. The autophagic process is involved in cellular development, differentiation, and apoptosis, and is also implicated in numerous human diseases, such as cancer and neurodegenerative disorders [19,20]. The discovery of autophagy-related genes in yeast has opened avenues for the application of numerous genetic approaches towards the mechanistic understanding of this cellular process. The molecular machinery of autophagy and its regulatory signaling pathways have been constructed in *S. cerevisiae*, and most mammalian orthologs have been identified as well. Autophagy is also required for the pathogenesis of several filamentous fungi, including *Magnaporthe oryzae*, *Cryptococcus neoformans*, *Aspergillus fumigatus* and *Candida albicans* [21]. *M. oryzae* is the most prominent fungal pathogen infecting cultivated rice. By attacking and forming an infectious appressorium on the aerial parts of plants, *M. oryzae* causes the eventual deterioration of plant growth and significant losses in rice yields approaching 30% [22]. Autophagy is required in the development of the infectious appressorium. In the process of infection, *M. oryzae* cells attach to plant leaves or stems and produce a penetration peg that can puncture the host cuticle; the fungus then invades the tissue via hyphal growth. The maturation of the appressorium is accompanied by autophagy-related cell death of the conidium, which allows cells to concentrate resources for growth into the host tissue rather than for surface-spread growth [23]. 

*C. neoformans*, *A. fumigatus* and *C. albicans* are all opportunistic human fungal pathogens. Each pathogen also utilizes autophagy to some degree in infection; however, the specific contributions of autophagy in each are distinct due to unique host-pathogen interaction mechanisms and growth physiologies. Autophagy is required for the virulence of *C. neoformans* but dispensable in the other two species [24,25,26]. *C. neoformans* primarily infects immunocompromised patients, especially those with impaired cellular immunity related to chemotherapy and HIV infection [27]. Significant levels of autophagy have been found during the infection process, and *C. neoformans VPS34* deletion and *ATG8* RNAi repression suppresses its virulence, supporting the existence of a linkage between autophagy and virulence in this pathogenic fungus [24]. 

### 2.2. The Molecular Machinery of Autophagy

**Figure 1:**
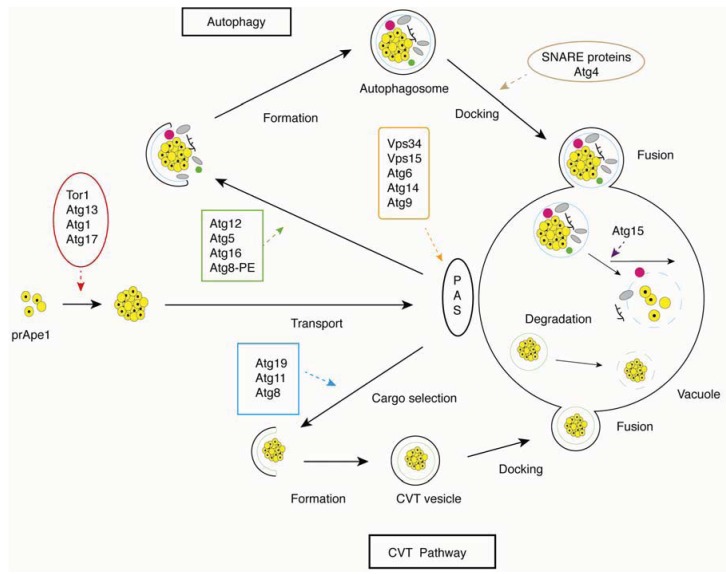
Overview of the basic steps in the autophagy and cytoplasm to vacuole (CVT) pathways in yeast. Critical steps are depicted graphically and indicated with arrows; proteins involved in a particular step are shown in boxes.

Autophagy is a complex multi-step process, with distinct sets of autophagy-related genes (*ATG* genes) mediating key steps from autophagic induction to vesicle fusion and autophagosome breakdown (Figure 1). Here, we present each of these steps separately, accompanied by an indication of genes associated with each sub-process.

#### 2.2.1. Autophagic Induction

Levels of autophagy are low under conditions of nutrient sufficiency but can be induced by inhibition of the serine/threonine protein kinase Tor (Target Of Rapamycin) [28], which acts as a crucial regulator of various stress response mechanisms under conditions of nitrogen stress. In this process, the inhibition of Tor directly or indirectly dephosphorylates Atg13p, and positively increases the kinase activity of another serine/threonine kinase Atg1p; Atg1p then binds Atg13p and Atg17p to form a multiprotein complex [29,30]. The Atg1p-Atg13p-Atg17p complex recruits multiple autophagy proteins to a site called the pre-autophagosomal structure (PAS) [31,32], where it is thought to play a critical role in autophagosome formation [33,34]. Another protein kinase Gcn2p and its phosphorylated substrate, the translation initiation factor eIF2α, can also induce autophagy under conditions of nitrogen stress [35]. 

#### 2.2.2. Cargo Selection and Packaging

Autophagy is either selective or non-selective. In *S. cerevisiae*, the cytoplasm to vacuole (Cvt) pathway is a selective autophagic process, and the protein components of this pathway provide a crucial transport route for the recognition and delivery of cargo to the vacuole [36,37]. In the Cvt pathway, a major cargo protein precursor aminopeptididase I (prApe1) is delivered to the vacuole to generate mature Ape1. The receptor protein Atg19p can recognize and bind to prApe1, promoting the formation of an Atg19p-prApe1 complex. Subsequent interaction with Atg11p then recruits the complex to the PAS, where Atg11p can interact with Atg8p, for packaging the receptor-cargo complex into Cvt vesicles [38,39].

#### 2.2.3. Autophagosome Formation

Distinct from vesicle formation throughout the endomembrane system, autophagic vesicles are not generated by budding from the surface of a preexisting organelle but instead are constructed at the PAS from newly generated membranes. The formation of the core and the construction of the new membrane require the class III phosphatidylinositol 3-kinase (PtdIns3K) complex, which includes the PtdIns 3-kinase Vps34p, Vps15p, Atg6p/Vps30p and Atg14p [40,41]. A second complex containing Vps15p, Vps34p, Atg6p and Vps38p participates in the delivery of vacuolar proteins through the Vps signaling pathway [42]. The activated PtdIns3K complex releases phosphatidylinositol 3-phosphate (PtdIns3P), which binds to PAS-targeting proteins, such as Atg18p, Atg20p, Atg21p and Atg24p [43,44,45]. 

Expansion of the autophagosome requires two ubiquitin-like (Ubl) conjugation systems, Atg12p-Atg5p-Atg16p and Atg8p-PE (phosphatidylethanolamine). Both Ubl proteins, Atg12p and Atg8p, can be activated by the E1 enzyme Atg7p. When activated, Atg12p is transferred to the E2 conjugating enzyme Atg10p and then binds to an internal lysine of its substrate protein Atg5p. Atg12p-Atg5p attaches to a coiled-coil protein Atg16p to form a multimeric complex [34,46]. The complex then associates with the autophagosome membrane via Atg5p. In the Atg8p-PE system, Atg8p is activated by Atg7p and is transferred to the E2 enzyme Atg3p; Atg8p is finally conjugated with the target lipid PE [47]. Both Atg12p-Atg5p-Atg16p and Atg8p-PE are required for decorating the expanding phagophore [46,48].

Aside from structural proteins, Atg9p is the only integral membrane protein involved in autophagosome formation. A population of Atg9p molecules is localized to the PAS site, while an additional population of Atg9p is found diffused throughout the cytosol. Transport of Atg9p between the PAS and non-PAS sites is necessary for autophagosome formation. Atg11p, Atg23p and Atg27p are responsible, although not solely so, for transporting Atg9p to the PAS structure [49,50], while the Atg1p complex, Atg2p and Atg18p are involved in its retrograde transport [51].

#### 2.2.4. Vesicle Docking and Fusion

Upon forming the autophagosome, the Atg12p-Atg5p-Atg16p complex is released back to the cytosol. However, Atg8p-PE follows the autophagosome to the vacuole, where Atg4p cleaves the complex, releasing Atg8p to the lysomsomal lumen for degradation [52]. Docking and fusion of the autophagosome to the vacuolar surface requires multiple SNARE proteins, Vam3p, Vam7p, Vit1p, Ykt6p, the Rab family GTPase Ypt7p, the NSF homolog Sec18p, and class C Vps/HOPS complex proteins [36].

#### 2.2.5. Autophagosome Breakdown

After fusion to the vacuole, the single membrane body of the autophagosome is broken down to recycle its cellular macromolecules. Two conserved components involved in breakdown of the autophagosome were identified in yeast, Atg15p and Atg22p. The lipase Atg15p, as well as proteinase A and B, are involved in degradation of the inner vesicle [53,54], while the integral vacuolar membrane protein Atg22p functions in transporting amino acids and other small molecules back to the cytosol for protein synthesis and maintenance of cellular functions during autophagy [55].

### 2.3. Filamentous Growth in Saccharomyces cerevisiae and Other Yeast Species

We review here the filamentous growth transition in *S. cerevisiae*, as it has been studied more intensely than corresponding modes of filamentous growth in other fungi [56]. Filamentous growth in budding yeast is induced by nutrient scarcity in the form of nitrogen stress or glucose deprivation. In addition, growth in the presence of short-chain alcohols stimulates filamentous growth; the short-chain alcohols are end products of amino acid catabolism under nitrogen-poor conditions and likely constitute a mimic of nitrogen stress, although distinctions exist regarding the genetic complement necessary for the induction of filamentous growth by each condition [57]. In general, the filamentous growth response is viewed as a foraging mechanism allowing non-motile yeast to scavenge for nutrients under stressful conditions.

During filamentous growth, yeast cells exhibit an elongated shape due to a delay in progression through G2/M, resulting in a prolonged period of apically directed polarized growth [9,58]. The budding pattern changes from axial (in haploid cells) or bipolar (in diploid cells) to a unipolar pattern [59]. Perhaps most strikingly, cells remain connected after cytokinesis during filamentous growth, forming a multicellular filament that resembles hyphal filaments observed in many fungi. Unlike true hyphal filaments, however, cells within the *S. cerevisiae* pseudohyphal filaments do not share cytoplasm, are not multinucleate, and lack parallel-sided cell walls [60]. This filamentous growth transition in *S. cerevisiae* has been studied extensively as a model of related pseudohyphal and hyphal growth transitions in the opportunistic human pathogen *C. albicans*, where the ability to transition between growth forms has been linked with virulence [61].

Many laboratory strains of *S. cerevisiae* are non-filamentous, and thus studies of filamentation are carried out in either the Σ1278b or SK1 genetic backgrounds. Though filamentous growth occurs in both haploid and diploid yeast cells, the morphological changes are slightly different between the two cell types. In a diploid strain of these backgrounds, nitrogen stress induces the formation of surface-spread filaments from a spotted culture or colony and invasive filaments that extend downward into a solid substrate below [62]. In a haploid strain, surface-spread filaments are much less extensive, but invasive filaments do form on both rich medium and under conditions of glucose deprivation [63]. Typically, the surface spread filamentation exhibited by a diploid strain under conditions of nitrogen stress is referred to as pseudohyphal growth. 

#### 2.3.1. The Genetic Basis of Filamentous Growth

Filamentous growth in *S. cerevisiae* is mediated by several signaling pathways. Classic studies from numerous laboratories have identified at least three signaling pathways that regulate filamentous growth: 1) the Kss1p MAPK pathway, 2) the Snf1p kinase pathway, and 3) the cAMP-responsive PKA pathway. The filamentous growth MAPK pathway is situated downstream of the P21-activated kinase Ste20p and consists of the MAPKKK Ste11p, the MAPKK Ste7p, and the MAPK itself Kss1p [64]. Kss1p regulates the key filamentous growth transcription factor Ste12p/Tec1p, which in turn activates expression of the flocculin *MUC1*/*FLO11* [65,66]. Snf1p is a member of the AMP-activated kinase family that regulates many processes, including the cellular response to glucose availability [67]. The PKA pathway will be discussed in a subsequent section of this review with respect to its role in regulating autophagy.

While these core signaling pathways play key roles in regulating the filamentous growth transition in *S. cerevisiae*, the full genetic basis of yeast filamentation is very broad. Large-scale phenotypic screens using transposon-mutagenized yeast strains have identified 309 genes that are required for wild-type filamentous growth in a haploid genetic background under conditions of butanol induction [10]. An overexpression screen in the same background identified 199 genes that yielded filamentous growth phenotypes [10], while microarray-based expression profiling studies have identified an extensive transcriptional program of 874 genes differentially expressed during filamentous growth [68]. The bulk of these genes are components of cellular processes required for wild-type filamentous growth. We survey a few of these underlying processes here.

#### 2.3.2. Cellular Processes Contributing to Yeast Filamentation

In order for yeast cells to effectively form pseudohyphal filaments, critical processes of budding, polarized growth, and cell cycle progression must be appropriately regulated and coordinated; of course, these processes are intimately related, and the molecular machinery of cell cycle progression and cellular morphogenesis has been reviewed recently in Howell and Lew [69]. With respect to filamentous growth, at least two genes required for wild-type bud site selection (e.g., *BUD2* and *BUD14*) yield filamentous growth phenotypes of defective surface filamentation upon gene disruption in a haploid genetic background under conditions of butanol treatment [10], and a larger set of genes involved in budding (including *BUD3*, *BUD4*, *BUD6*, *BUD7*, *BUD8*, and *BUD25*) yield deletion-based filamentous growth defects in either haploid invasive growth, diploid pseudohyphal filamentation, or biofilm formation. Similarly, in addition to *BUD6*, key polarisome components such as *SPA2* and *PEA2* also yield defects in surface filamentation in a haploid strain upon butanol treatment [10]. Genes that regulate the G2/M transition in yeast also affect pseudohyphal growth, leading to the general observation that genetic perturbations resulting in delayed G2/M progression and an extended period of apical growth promote yeast filamentation. This generalization must be considered with caution, however, as many genetic perturbations that affect cell cycle regulators modulate events that impact processes outside of G2/M progression; consequently, it can be difficult to predict the resulting filamentous growth phenotype in such mutant strains.

#### 2.3.3. Downstream Genes Mediating Increased Cell-Cell Adhesion

The enhanced cell-cell adhesion of cells undergoing filamentous growth is a well-established hallmark of the process. Genetic studies have identified a set of genes required for calcium-dependent aggregation, or flocculation, in yeast, and many of these genes contribute to the yeast filamentous growth response, particularly in response to conditions of nitrogen stress. These flocculation genes comprise a multi-gene family of largely sub-telomeric sequences encoding several lectin-like proteins, with *FLO8* and *MUC1/FLO11* being most critically associated with filamentous growth. Flo8p is a transcription factor that regulates the expression of *MUC1* and many other genes contributing to filamentous growth; approximately 230 such gene promoters bound by Flo8p have been identified through chromatin immunoprecipitation-microarray analyses [70]. Interestingly, deletion of *FLO8* yields a strong filamentous growth defect, and the *FLO8* sequence is in fact a pseudogene in strains derived from the common non-filamentous S288c genetic background. Flo8p is discussed again as a downstream effector of PKA signaling later in this review. *MUC1* has been studied most intensively for its large promoter, which integrates transcriptional signals from Flo8p, Ste12p/Tec1p and Mss11p [71]. Muc1p is a GPI-anchored cell surface glycoprotein required for invasive growth, pseudohyphal formation, and biofilm formation. It is also discussed in this review with respect to PKA signaling and as a downstream effector regulated by the filamentous growth MAPK Kss1p pathway. 

#### 2.3.4. An Interrelationship Between Autophagy-Related Genes and Yeast Filamentation

Interestingly, some autophagy-related genes may contribute to the yeast filamentous growth transition as well (Table 1). Among the 30 autophagy-related genes identified in yeast, 14 have been found by microarray-based expression profiling to be transcriptionally induced during filamentous growth [17]. Of course, the significance of this observation is unclear, as nitrogen stress is a common induction mechanism for both processes. More substantially, the overexpression of 10 autophagy-related genes (*ATG1*, *ATG3*, *ATG4*, *ATG6*, *ATG7*, *ATG17*, *ATG19*, *ATG23*, *ATG24*, and *ATG29*) inhibits filamentous growth. Consistent with these results, the deletion of *ATG1* and *ATG7* results in exaggerated filamentous growth and the premature initiation of filamentous growth under less severe conditions of nitrogen stress [16]. As a simple model to consider these results, we proposed previously that the inhibition of autophagy resulting from deletion of *ATG1* and *ATG7* results in an increased degree of nitrogen stress, manifested as exaggerated filamentous growth. However, it is unclear whether the overexpression of individual autophagy-related genes is sufficient to generate an overall increase in the level of autophagic activity; consequently, the overexpression results are not as easily explained through a physiological connection and hint at additional regulatory interconnections between the processes. 

The deletion and overexpression results suggest an antagonistic relationship between autophagy and yeast filamentous growth; this was an unexpected finding, but one that can be reconciled with the functions of each process in the yeast response to nitrogen stress. Autophagy may be rapidly responsive to nitrogen stress, acting to dampen the filamentous growth response until nitrogen limitation becomes more severe, at which point signals activating filamentous growth may be sufficiently strong to initiate the process. At present, this model is consistent with genetic data from analyses of autophagy-related genes, but nonetheless speculative in that relevant experiments to conclusively prove or disprove the model remain to be undertaken. The molecular mechanisms regulating the interconnections between autophagy and filamentous growth also remain to be elucidated. The genetic analyses described above suggest a potential regulatory connection between autophagy-related genes (e.g., *ATG1*) and filamentous growth regulators; however, to date no such connections with Atg1p or other autophagy-related genes are evident, even from large-scale mass spectrometry studies or transcriptomics. Although this type of direct regulatory connection remains to be identified, several signaling pathways are known to regulate both processes consistent with the indicated deletion and overexpression studies, and the following sections highlight several such signaling pathways as candidate molecular links between autophagy and yeast filamentous growth.

**Table 1:** Summary of autophagy-related genes with potential contributions to filamentous growth.

Gene	Autophagy Process	Protein Function/Description	Transcript Levels in Early Fil. Growth	Deletion (Δ)/Overexpression (OE) Phenotype
*ATG1*	Induction; retrieval	Protein kinase	Increased	Decreased fil. growth (OE); Exaggerated fil. growth (Δ)
*ATG3*	Vesicle expansion and completion	Conjugation enzyme	Increased	Decreased fil. growth (OE)
*ATG4*	Vesicle expansion and completion	Cysteine protease	Increased	Wild-type fil. growth (OE)
*ATG5*	Vesicle expansion and completion	Conjugation enzyme	Increased	Untested
*ATG6*	Vesicle nucleation	PI3P binding	Increased	Wild-type fil. growth (OE)
*ATG7*	Vesicle expansion and completion	Activating enzyme	Increased	Decreased fil. growth (OE); Exaggerated fil. growth (Δ)
*ATG8*	Vesicle expansion/completion	Ubiquitin-like protein	Increased	Untested
*ATG9*	Vesicle nucleation; retrieval	Integral membrane protein	Increased	Untested
*ATG14*	Vesicle nucleation	PI3-Kinase complex	Increased	Untested
*ATG17*	Induction	Atg1p modulator	Increased	Decreased fil. growth (OE)
*ATG19*	Induction	PI3P binding	Increased	Decreased fil. growth (OE)
*ATG20*	Induction	PI3P binding	Increased	Untested
*ATG21*	Cvt pathway	PI3P binding	Increased	Untested
*ATG22*	Efflux from the vacuole	Vacuolar permease	Increased	Untested
*ATG23*	Cvt pathway	PI3P binding	Unchanged	Decreased fil. growth (OE)
*ATG24*	Autophagic body breakdown	Vacuolar membrane protein	Unchanged	Decreased fil. growth (OE)
*ATG29*	Peroxisome sequestration	UDP-glucose	Unchanged	Decreased fil. growth (OE)

### 2.4. Autophagy and Filamentous Growth Signaling Pathways

Several signaling pathways are known to contribute to both wild-type autophagy and filamentous growth. We review below the TORC1, PKA, and Snf1p signaling pathways, highlighting potential effects on both of these cellular processes.

#### 2.4.1. Tor Complex 1

As mentioned above, Tor is a critical regulator of both autophagy and the Cvt signaling pathway in *S. cerevisiae*. The Tor kinase functions in two distinct multiprotein complexes, TORC1 (Tor complex I) and TORC2 (Tor complex 2). Of these complexes, TORC1 fulfills a primary role in regulating autophagy [12]. Under conditions of nutrient sufficiency, Atg13p is hyperphosophorylated by TORC1 directly or indirectly, resulting in its low affinity with Atg1p and Atg17p. In this case, diminished formation of the Atg1p-Atg13p-Atg17p complex inhibits autophagy [29]. From the observation that Atg17p can bind to Atg13p in the absence of Atg1p but cannot bind to Atg1p without Atg13p, Atg13p seems to mediate the interaction between Atg1p and Atg17p [72]. In contrast, the inhibition of TORC1 by rapamycin treatment or nitrogen starvation reduces the phosphorylation state of Atg13p. Hypophosphorylated Atg13p mediates the interaction of Atg1p and Atg17p to form the Atg1p-Atg13p-Atg17p complex. Interaction with Atg13p elevates Atg1p kinase activity, which is crucial in autophagy and the Cvt pathway. Recent studies have shown that the kinase activity of Atg1p is required for re-phosphorylation of Atg13p and the recovery of TORC1 activity during prolonged nitrogen starvation [73]. It is unclear whether the kinase activity of Atg1p or its structural change actually plays the essential role in inducing autophagy, as a critical downstream substrate of Atg1p during autophagic induction has not been identified.

**Figure 2:**
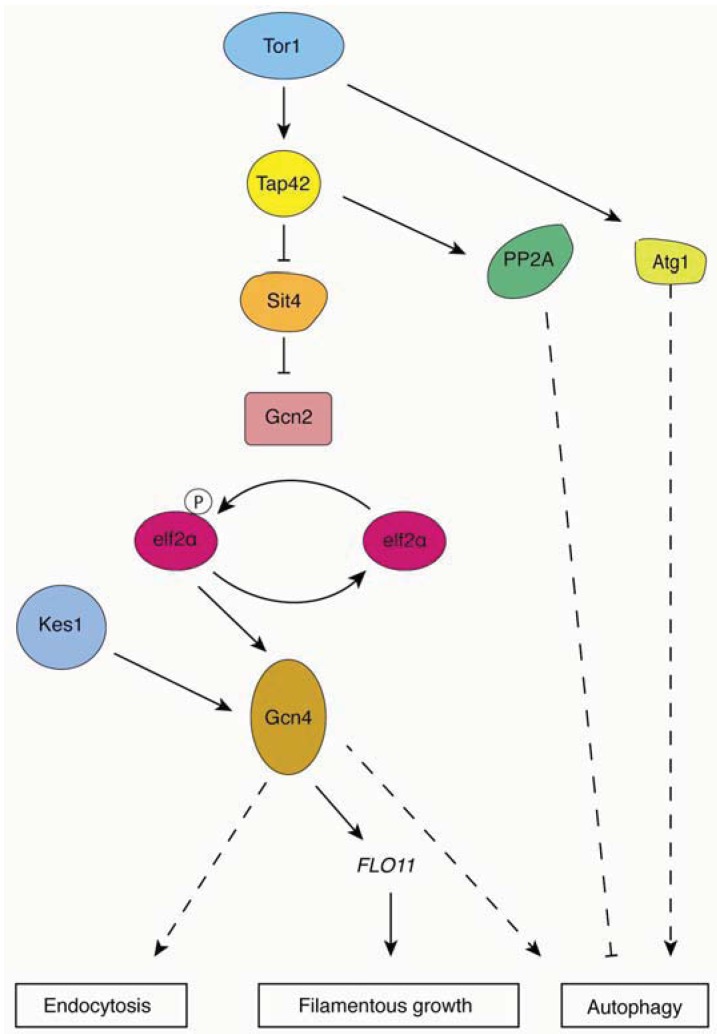
Overview of TORC1 signaling pathways. Relevant regulatory connections are highlighted contributing to yeast pseudohyphal growth, autophagy, and endocytosis. Dashed lines indicate effects that encompass additional unlisted proteins.

In addition to regulating the Atg1p-Atg13p-Atg17p complex, TORC1 also acts through its downstream effectors, particularly the serine/threonine phosphatase type 2A (PP2A) and 2A-related protein phosphatase Sit4p, and Tap42p [74] (Figure 2). TORC1 inhibits autophagy by directly phosphorylating Tap42p, or by indirectly phosphorylating Tap42p through Tap41p. Under conditions of nitrogen stress, the dephosphorylated form of Tap42p interacts with the catalytic subunits of PP2A and Sit4p [75]. Recent studies indicate that inactivation of PP2A induces autophagy and that overexpression of the PP2A catalytic subunit inhibits autophagy, suggesting that PP2A is a negative regulator in the Tor pathway [76]. Although the downstream target of PP2A is unknown, it is widely believed that Tap42p transmits a signal from TORC1 to regulate autophagy. 

Another possible target of TORC1 is the protein kinase Gcn2p. Rapamycin-activated Gcn2p phosphorylates its only known substrate eIF2α, resulting in activation of the transcriptional transactivator Gcn4p [77]. Both *GCN2* and *GCN4* deletion mutants and the eIF2α kinase dead mutant eIF2α *SUI2*^S51A^ fail to induce autophagy under conditions of nitrogen starvation, confirming the role of eIF2α kinase signaling in regulating autophagy [35].

There are two mechanisms through which Tor signaling regulates filamentous growth under conditions of nitrogen stress. First, TORC1 regulates transcript levels of *MUC1*/*FLO11* via the transcription factor Gcn4p. It has been shown that expression levels of a *FLO11-LacZ* reporter are significantly decreased in a *gcn4*Δ mutant, while overexpression of *GCN4* is sufficient to induce filamentous growth and elevated *FLO11* expression [13]. These results suggest that Gcn4p contributes to the transcriptional regulation of *FLO11*. The transcription factor Flo8p is also a known regulator of *MUC1*/*FLO11* expression [14]. Second, recent studies have identified multiple proteins associated with the Kog1p/TORC1 complex, such as Mks1p, Kap123p, Hef3p, Ksp1p and Uba3p [78]. Deletion mutants of *KSP1*, *KAP123* and *UBA4* exhibit impaired invasive growth, and the diploid deletion strains show reduced spread filamentation compared to a wild-type strain [79]. The fact that *FLO11* transcript levels are reduced in these mutants provides more evidence that TORC1 does indeed contribute to the regulation of *MUC1*. Furthermore, the proteins Tap42p and Sit4p are known to regulate autophagy and have also been found to yield filamentous growth phenotypes upon genetic perturbation. Overexpression of *TAP42* restores filamentous growth in cells treated with rapamycin, and deletion of the Sit4p phosphatase impairs filamentous growth and shows rapamycin hyperactivity [80]. These results indicate a model wherein TORC1 changes the association of Tap42p and Sit4p by phosphorylating Tap42p to regulate filamentous growth in yeast.

#### 2.4.2. The Ras/PKA Pathway

In addition to TORC1, the Ras/PKA pathway is another conserved signaling network regulating autophagy in yeast and mammals [15,81]. Yeast PKA contains one regulatory subunit, Bcy1p, and three catalytic subunits, Tpk1p, Tpk2p and Tpk3p. Under conditions of nutrient sufficiency, two redundant small GTPases, Ras1p and Ras2p, are activated by upstream signals and then stimulate adenylyl cyclase to enhance cAMP levels in the cell. cAMP binds to the PKA regulatory subunit Bcy1p to release its catalytic subunits Tpk1p, Tpk2p and Tpk3p, resulting in activation of PKA. In addition to TORC1, PKA is an essential negative regulator of autophagy. The activation of PKA phosphorylates Atg1p, causing it to dissociate from the PAS [82]. It should be noted that multiple Atg proteins contain PKA phosphorylation sites, so Atg1p may not serve as the only protein substrate downstream of PKA. Another possible substrate of PKA is Atg13p. It has been shown that PKA directly phosphorylates Atg13p, and PKA phosphorylation regulates the association of Atg13p with the PAS. Constitutive activation of PKA through hyperactivation of RAS, as observed in the *RAS^G19^V* allele, suppresses autophagy induced by Tor under nitrogen starvation or rapamycin induction [83]. This result suggests that the Ras/PKA pathway may function downstream of the Tor signaling pathway. 

**Figure 3:**
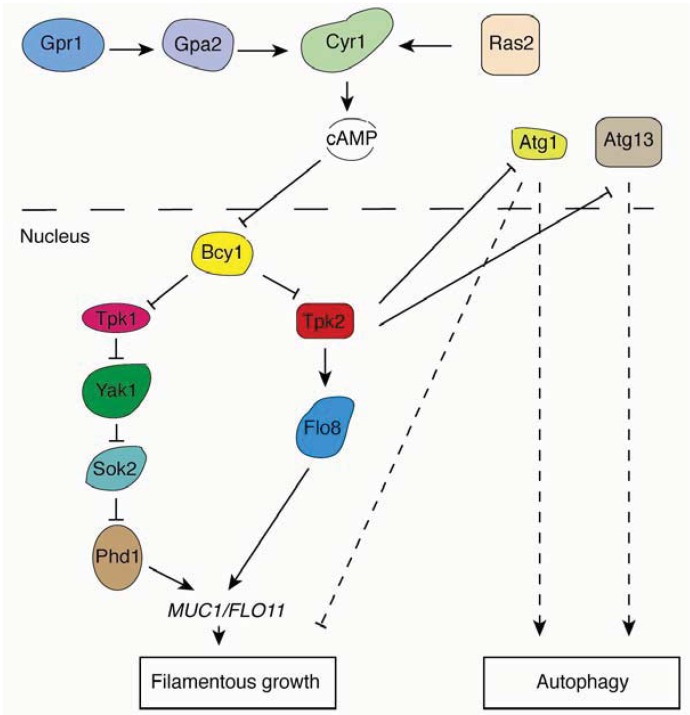
Overview of the Ras/PKA pathway and relevant regulatory connections involved in the yeast pseudohyphal growth and autophagy responses.

The Ras/PKA pathway also plays a critical role in regulating filamentous growth in the budding yeast. Interestingly, the functions of the PKA catalytic subunits Tpk1p, Tpk2p, and Tpk3p are distinct from each other in regulating filamentous growth. Whereas deletion of *TPK2* impairs filamentous growth, the deletion of *TPK1* and *TPK3* actually enhances filamentous growth [14]. These results suggest that Tpk2p is an activator of filamentous growth, while Tpk1p and Tpk3p may possess an inhibitory function. The key filamentous growth transcription factor Flo8p is a direct substrate of Tpk2p, and deletion of *FLO8* abolishes filamentous growth in the Σ1278b genetic background. *FLO8* is an essential regulator of filamentous growth; in fact, many non-filamentous strains contain a premature stop codon in the *FLO8* coding sequence, rendering the open reading frame a non-functional pseudogene in these strains (e.g., S288c derivatives) [84]. Relevant regulatory relationships associated with the Ras/PKA pathway are illustrated in Figure 3. 

In addition to PKA, the yeast ortholog of mammalian PBK/Akt, Sch9p, is a negative regulator of autophagy. Inactivation of both Sch9p and PKA induces autophagy under conditions of nutrient-sufficiency, and the inactivation of TORC1 can contribute further to this effect [81]. Thus, Sch9p, PKA and TORC1 may regulate autophagy in parallel. It is suggested that Sch9p also functions downstream of the Tor signaling pathway because it is a direct substrate of TORC1. But, to date, the mechanism by which TORC1 regulates Ras/PKA and Sch9p remains unclear.

#### 2.4.3. The Nutrient-Sensing Snf1p Pathway

Snf1p, a cAMP-activated protein kinase required for glucose derepression is also a positive regulator of autophagy. Snf1p mutants fail to induce autophagy under conditions of nutrient starvation. The overexpression of *ATG1* and *ATG13* increases stationary-phase glycogen accumulation in *snf1*Δ cells, indicating that Snf1p may act upstream of Atg1p and Atg13p [85]. 

In addition to nitrogen stress, glucose deprivation is known to act as another trigger of filamentous growth. Several studies have shown that the depletion of fermentable carbon sources like sucrose and glucose can trigger filamentous growth [63,86]. Snf1p is known to regulate filamentous growth through its beta subunit Gal83p. Snf1p-Gal83p antagonizes the zinc-finger proteins Ngr1p and Ngr2p to de-repress transcription of *MUC1*/*FLO11* [67]. Snf1p also acts to regulate filamentous growth through another beta subunit, Sip2p, as well as other substrates [67,87].

### 2.5. TORC1 Regulates Endocytosis in S. cerevisiae

Endocytosis is an important cellular process by which cells can uptake extracellular components such as fluid, proteins, and even large particles, by trafficking them along with the plasma membrane to a central vacuole or lysosome. Importantly, during endocytosis, plasma membrane lipids and proteins are remodeled to ensure appropriate membrane composition. Increasing evidence from studies involving a number of cell types has highlighted the importance of the actin cytoskeleton during endocytosis. The discovery that a sub-apical collar of endocytic actin patches exist behind the growing hyphal apex raises the notion that endocytosis may be related to hyphal growth in filamentous fungi [88]. Evidence in support of this possibility includes the functions of actin and actin-binding proteins, which recycle the apical membrane and modulate the cytoskeletal structure at the hyphal tip in the filamentous fungi *Aspergillus nidulans* and *Neurospora crassa* [89,90]. Higuchi *et al*. also found that *AOEND4*, an ortholog of the endocytosis-related *END4/SLA2* gene of *S. cerevisiae*, plays a vital role in hyphal tip growth in *Aspergillus oryzae* by regulating cell wall synthesis [91]. 

Tor is a key regulator of cell growth and metabolism in response to environmental changes, and Tor-mediated signaling pathways are conserved in yeast and mammals [92]. Recent studies reveal that in addition to autophagy and filamentous growth, endocytosis is also affected by TORC1 function; specifically, deletion of the TORC1 subunit Tco89p inhibits the function of Can1p, an arginine transporter that mediates endocytosis. Interestingly, Can1p in yeast localizes to an ergosterol-rich domain in the plasma membrane, the so-called membrane compartment of Can1p or MCC [93]. The MCC houses several proteins that are required for filamentous growth, including the uracil permease Fur4p and the MCC component protein Pun1p [89]. MacGum *et al*. also identified that TORC1 inhibits activity of the protein kinase Npr1p, which negatively regulates endocytosis by phosphoinhibiting the arrestin-like adaptor protein Art1p and promoting Art1p-Rsp5p translocation to the plasma membrane [94].

TORC1 is a common regulator of endocytosis, autophagy, and filamentous growth, but what other proteins may also contribute to the regulation of this set of cellular processes? Possibly, such connections may be mediated by PtdIns-4-P, the sterol-binding protein Kes1p, and the transcription factor Gcn4. Mousley *et al*. have shown that Kes1p is required in membrane and lipid trafficking through the trans-Golgi network and endosomal systems. A heterozygous diploid *KES1*/*kes*1^Y97F^ mutant strain shows impaired trafficking to the plasma membrane and is unable to induce autophagy under conditions of NH4^+^ starvation, suggesting dual roles for Kes1p in endocytosis and autophagy. In addition, enhanced activity of Kes1p can release the transcriptional derepression of *GCN4*, which encodes a well-studied transcriptional factor involved in the general amino acid control (GAAC) pathway [95]. This result suggests that Kes1p may also mediate filamentous growth via Gcn4p. 

## 3. Conclusions

In this review, we have explored the interconnections among autophagy, filamentous growth and endocytosis. Recent studies have demonstrated that under conditions of nitrogen stress in yeast, these three metabolic processes are regulated by the same key components and possibly intervene in similar signaling pathways. The amino acid sensor TORC1 is a crucial upstream regulator, opening the downstream signaling cascade to modulate cellular catabolism in adaptation to conditions of nutrient starvation. Key components, including Kes1p, Atg1p, Gcn4p, and Sit4p possess multiple functions contributing to the regulation of two, or even all three, mechanisms in this process. The RAS/PKA pathway is another signaling module shared by autophagy and filamentous growth, wherein the PKA subunits Tpk1p and Tpk2p control transcriptional levels of the downstream genes involved in both autophagy and filamentous growth. Genetic evidence from the perturbation of autophagy-related genes highlight an antagonistic interplay between autophagy and filamentous growth, with the TOR and cAMP-PKA pathways potentially providing the molecular link between the processes. Both the TOR and PKA pathways negatively regulate autophagy, while positively regulating the yeast filamentous growth response. Interestingly, the relationship between autophagy and filamentous growth may be less clear in pathogenic fungi, as evidence exists that autophagy and filamentation/hyphal development are both required for virulence. It is possible that the antagonistic relationship between both processes may still exist in these pathogens, but that perturbation of the life cycle/morphology resulting from inhibition of either process is sufficient to interfere with critical steps in infection.

The relationship between autophagy, filamentous growth, and endocytosis is likely less direct than the link between autophagy and filamentous growth. In this review, we discuss some of the genes in common between the pathways that regulate these processes. In particular, we expect that the TOR pathway is the critical link between all three processes. Furthermore, we speculate that the link between filamentous growth and endocytosis likely stems from the fact that endocytosis modulates the activity of transporters that are required for the filamentous growth response.

The connection between autophagy and filamentous growth is quite interesting. The experimental data summarized here suggests an interrelationship between these nitrogen stress-responsive processes with respect to the timing and degree of initiating stimulus. We speculate that autophagy may be ongoing at a low level and/or rapidly initiated in response to low levels of nitrogen stress. Yeast cells may be able to efficiently respond to low-level nitrogen stress through autophagy, while only initiating the complex program of filamentation in response to more severe levels of nitrogen limitation. By this model, some autophagy-related proteins (e.g., the kinase Atg1p) may dampen or down-regulate filamentous growth until sufficient activating signals from multiple pathways act to override inhibitory signals from autophagy-related genes and other sources. Filamentous growth certainly represents a significant change in cellular morphology and properties, such that cells may not be cost-effective in initiating such a process under mild stress conditions. At present, this model is highly speculative, and, with that in mind, we present several candidate pathways with observed functional effects on both processes consistent with this model. Of course, the molecular overlap among these signaling components is not deterministic for supporting the idea that autophagy, filamentous growth and endocytosis are interrelated in *S. cerevisiae*. Indeed, more evidence will be needed in order to conclusively unveil the interplay between these important cellular processes.
